# Preliminary Efficacy of a Self‐Management Programme to Improve Quality‐of‐Life in Patients With Obesity and Osteoarthritis Awaiting Arthroplasty: A Randomised Trial

**DOI:** 10.1111/ijn.70044

**Published:** 2025-08-21

**Authors:** Ladan Sahafi, David Smith, Ruurd Jaarsma, Malcolm Battersby

**Affiliations:** ^1^ College of Medicine and Public Health Flinders University Adelaide Australia; ^2^ Department of Psychiatry, College of Medicine and Public Health Flinders University Bedford Park South Australia Australia

**Keywords:** arthroplasty, nurse‐led, obesity, osteoarthritis, quality of life, self‐management

## Abstract

**Background:**

Obesity is a risk factor for osteoarthritis and total hip/knee joint replacement and can lead to poorer outcomes following surgical interventions.

**Aim:**

This work aimed to determine the preliminary efficacy of a self‐management programme versus usual care in improving health‐related quality of life in obese patients with osteoarthritis awaiting joint replacement.

**Methods:**

This was a two‐group parallel randomised trial involving patients with obesity and osteoarthritis who were awaiting hip or knee arthroplasty. Patients were randomly allocated to the Flinders Program of self‐management support plus usual care or usual care alone groups. Primary outcomes at 10 months were Short‐Form Health Survey (SF‐36) and Osteoarthritis of Knee/Hip Quality of Life (OAKHQoL).

**Results:**

Ninety‐five patients were randomised to either intervention (*n* = 48) or usual care (*n* = 47) and analysed in an intent‐to‐treat analysis. While there was no intervention effect in SF‐36, evidence was in favour of intervention for OAKQoL improved social support (*d* = 0.43, 95% CI: 0.01–0.83) versus usual care (*d* = −0.01, 95% CI: −0.41 to 0.42) (*p* = 0.03). Similarly, intervention patients experienced larger improvements for social activity (*d* = 0.47; 95% CI: 0.05–0.89) versus usual care (*d* = −0.16; 95% CI: −0.58 to 0.25) (*p* = 0.005).

**Conclusion:**

The intervention warrants examination in a larger trial to establish effectiveness among patients with obesity and osteoarthritis awaiting arthroplasty.

**Trial Registration:** Australian New Zealand Clinical Trials Registry ACTRN12615000674538

## Summary

What is already known about this topic?

- Obesity comorbid with osteoarthritis (OA) is associated with high complication rates post‐arthroplasty.
- The role of nurses encountering OA is essential in providing management in primary care and specialty settings.
- Self‐management programmes show promise in a range of chronic illnesses, but evidence is limited for OA.

What this paper adds:

- Preliminary evidence from a randomised trial suggests that the self‐management intervention may have efficacy for improving mental and social domains among patients.
- Findings also provide evidence that a nurse‐led self‐management intervention for patients with obesity and osteoarthritis awaiting arthroplasty is feasible.

The implications of this paper:

- Nurses are ideally positioned to provide key aspects of self‐management intervention for patients with comorbid obesity and osteoarthritis as they are a core member of the joint replacement assessment team, are trusted by patients and are aware of psychosocial factors which impact on a patient's motivation and adherence to medical and nonmedical interventions prior to surgery.
- The intervention warrants examination in a larger multi‐site trial to establish efficacy/effectiveness in a heterogeneous population and help inform policy and practice.

## 1 Introduction

In Australia, approximately 3.20 million people have been estimated to have osteoarthritis (OA), with substantial growth in the prevalence of different types including hip (+171%) and knee (+126%) from 1990 to 2019 (Ackerman et al. 2022). The proportion of hip and knee OA burden attributable to obesity has been estimated as 12% and 36%, respectively, in 2019 and has led to a high risk of hip and knee arthroplasty (total hip/knee replacement) (Ackerman et al. 2022; Chen et al. 2022). As life expectancy increases, the number of people living with OA for long periods is expected to grow (Australian Institute of Health Welfare 2019). Co‐occurring obesity and OA traps individuals in a vicious cycle (Antonelli and Starz 2012). Obesity increases the progression of OA as well as joint loading. This results in diminished exercise capability and reduced muscle strength, which further increases both joint problems and obesity (Bliddal and Christensen 2006). Arthroplasty is the most practical treatment for end‐stage hip/knee OA patients suffering extensive pain and deformity when other treatments have failed (Munugoda et al. 2022). However, obesity comorbid with OA is associated with high complication rates post‐arthroplasty, including infection (Australian Institute of Health Welfare 2019).

The roles of nurses encountering OA are essential in providing management in primary care and specialty settings including teaching, research and other tasks and settings (Goh et al. 2006; Hill 2007; Juhola et al. 2007; Chao and Kalunian 2010; Antonelli and Starz 2012). This includes advocating weight loss for obese individuals with hip or knee OA and incorporating self‐management support (Antonelli and Starz 2012; Huffman et al. 2024). A previous Cochrane review of educational and self‐management interventions to guide nursing practice highlighted their benefits for patients suffering from asthma and also as promising interventions in areas such as diabetes mellitus, epilepsy and mental health (Coster and Norman 2009). While self‐management programmes are increasingly used in the management of chronic conditions (Lorig and Holman 2003; Coleman et al. 2010; McKnight et al. 2010; Jolly et al. 2018), evidence for their effectiveness in obese hip/knee OA patients awaiting arthroplasty is limited (Buchbinder et al. 2014).

Of nine RCTs evaluating the effectiveness of self‐management programmes in hip/knee OA patients to date, four showed significant improvements in patients who received self‐management support compared with the control group (Hurley et al. 2007; Coleman et al. 2012; Bennell et al. 2016; Kwok et al. 2016). Of these, one showed large beneficial effects, and three indicated small beneficial effects (Hurley et al. 2007; Coleman et al. 2012; Bennell et al. 2016). However, awaiting arthroplasty was not a primary focus in any of these trials alongside comorbid obesity. One previous study has used the Flinders self‐management support programme (FP) in hip/knee OA patients awaiting nonurgent arthroplasty and found statistically nonsignificant differences in health‐related quality of life (HRQoL) between the two groups (Crotty et al. 2009). A shortcoming of this study was that only 52% of participants completed their programme, and the influence of obesity on outcomes was not considered.

Self‐management support programmes are either disease‐specific patient education programmes or lay‐led group programmes, the most well‐known being the Stanford Arthritis Self‐Management Program (Lorig 1982). Disease‐specific programmes provide education which is often factual with less focus on acquiring skills and are usually delivered by health professionals. Lay‐led group programmes aim to improve participants' confidence in managing their chronic conditions, in partnership with health professionals. A major differentiation of the Flinders Program is that it is generic, that is, focusses on any chronic disease(s) or multimorbidities in the same person and is a health practitioner delivered individual care planning approach, tailored to the needs of patients including to maintain a reasonable quality of life (Battersby et al. 2015). The development of the programme was motivated by a trial (Council of Australian Governments [COAG] National Coordinated Care Trials) conducted to improve the health outcomes and treatment options for patients with chronic and complex health conditions. A midtrial review found that health benefits from coordinated care depended more on patients' self‐management behaviour than severity of illness (Battersby et al. 2007). In subsequent studies, the Flinders Program has been successfully delivered by nurse practitioners and allied‐health professionals at patients' homes, health services and by phone (Battersby et al. 2013; Battersby et al. 2015).

To date, self‐management programmes have shown some promise in benefiting OA patients, though the evidence base still remains at a nascent stage (e.g., (Uritani et al. 2021; Wu et al. 2022; Zhang et al. 2025). In terms of the most recent interventional RCTs for OA, arthroplasty and obesity nexus, findings from a UK feasibility study involving a behavioural change package including weight loss in patients awaiting knee replacement surgery supported progression to a large‐scale effectiveness trial (Simpson et al. 2024). To help further build the research trajectory, the primary aim of this current Australian‐based study was to obtain preliminary efficacy estimates of a nurse‐led FP for patients with obesity and OA awaiting hip/knee arthroplasty, using a randomised clinical trial design. The secondary aim was to assess feasibility based on key trial parameters.

## 2 Methods

### 2.1 Aims

The primary aim was to determine if there was a clinically meaningful change (improvement) in HRQoL scores across time (baseline to 10‐month follow‐up) for obese OA patients awaiting hip or knee replacement surgery who receive the Flinders Program plus usual care versus usual care alone. The secondary aim was to determine if study procedures were feasible.

### 2.2 Design

To address study aims, a two‐group randomised, parallel design, with outcomes assessed up to 10 months post‐randomisation, was employed. Primary outcomes were measures of generic and disease‐specific HRQoL. Feasibility was assessed using key trial parameters: screening, eligibility, consent, randomisation, retention, completion, missing data and intervention adherence rates. Additional findings from qualitative interviews to explore patient experiences of the trial will be reported in a separate publication. The study rationale and protocol have been published elsewhere (reference masked for review). We used the Consolidated Standards of Reporting Trials (CONSORT) for parallel group randomised trials to report study findings (Moher et al. 2010).

### 2.3 Participants

The trial was conducted in Adelaide, Australia. Participants were recruited from the Repatriation General Hospital between July 2015 and February 2016. We identified consecutive patients with obesity and OA from the hip or knee replacement waiting list and sent them an invitation letter to participate in the study. A week later, we contacted each patient by phone to ascertain their willingness to participate in the study. For those interested in participating, a visit time at the hospital was arranged to obtain their written consent and then administer baseline measures. Patients were eligible for the study if they had a BMI of 30 kg/m^2^ or above and were on a hip/knee replacement waiting list due to OA. Patients were ineligible if they had dementia or had surgery within the past 3 months and if they had an emotional or neurological condition that would prevent their ability or willingness to participate in the study.

### 2.4 Intervention

Patients were randomly allocated to one of two groups, the FP (the intervention) plus usual care or usual care alone (control). The FP was delivered by an orthopaedic nurse who was trained to administer the programme. The FP uses the self‐administered Partners in Health (PIH) scale to assess self‐management, the clinician conducted Cue and Response interview to identify self‐management strengths and barriers, and Problems and Goals assessment to formulate psychosocial goals. These components contribute to an individual care plan comprising self‐management and medical issues, patient aims, interventions, responsibilities and review dates. The PIH has been validated in various populations (Smith et al. 2017). Issues of diet, exercise and self‐management skills (dealing with everyday tasks, self‐monitoring and pain coping) were included in the care plan. The first assessment session was conducted face to face at the Repatriation General Hospital; then the participants were provided with two to four weekly phone call follow‐ups by the same nurse for up to 6 months to monitor progress and provide feedback and motivation.

The usual care (control) programme involved a one‐hour group information session about what to expect after surgery and how to accelerate recovery by committing to the provided exercises. Patients were also informed about healthy weight, setting weight loss goals as well as resources to advise on pain relief medications and supplements. An expert from Arthritis Australia then provided information about exercises before and after surgery. After the information session, patients were contacted on the phone once or twice by nurses to provide general support.

### 2.5 Randomisation and Blinding

Eligible patients were randomly assigned to one of two study groups following baseline assessment with a 1:1 allocation ratio. Randomisation was blocked and stratified by gender and BMI groups. BMI groups were defined as 30–34.9 (obese), 35–39.9 (severely obese) and 40 and above (morbidly obese). A biostatistician independently generated random sequences for each stratum using Stata version 16.1 software (StataCorp 2019), and the sequences were concealed and recorded in a separate database accessed from a separate computer. A staff member independent of the trial was responsible for assigning patients to random allocation groups. Staff enrolling and referring participants, collecting and entering data and administering interventions did not know in advance which treatment the next participant would receive. As all participants were informed that the intervention was a self‐management support programme, they were aware of whether they were receiving such a programme. Participants were advised not to discuss their allocation with the research assistant (author initials) who performed the data collection. The clinician delivering the intervention programme was unblinded. Data analysis was performed by (author initials) who was blinded to treatment allocation by noninformative labels for group variables.

### 2.6 Data Collection

Descriptive measures used to assess the feasibility of key trial parameters were focussed on participant study flow: screening, eligibility, consent, randomisation, retention, completion, missing data and intervention adherence rates.

The primary outcome measure was HRQoL administered at baseline, 6‐month and 10‐month follow‐up visits. To assess HRQoL, both generic and disease‐specific instruments were used. A generic measure was chosen for broad application across an individual's general health status, while an OA‐specific measure was designed to assess particular impacts of the disease over time with treatment, which is not possible with generic measures (Wells et al. 2011). Though some concepts addressed by the generic and disease‐specific instruments may overlap, their sensitivity to treatment may differ (Patrick and Deyo 1989). Using both generic and specific instruments in this current early phase trial provides an opportunity to appraise their sensitivity and feasibility for future larger‐scale trials.

The 36‐item Short‐Form Health Survey (SF‐36) questionnaire was used to measure generic HRQoL and Osteoarthritis of Knee/Hip Quality of Life (OAKHQoL) to measure OA disease‐specific HRQoL. The SF‐36 is a self‐administered measure of health perception in the general population, covering three main areas (functional status, well‐being, overall evaluation of health) using eight multi‐item domains: role limitations due to physical problems; bodily pain; general health perception; vitality; social limitations owing to emotional problems; role limitations due to emotional problems; and mental health, with these summarised in two component scores, physical and mental. The instrument has been shown to possess good acceptability by patients, structural validity and reliability/internal consistency (Brazier et al. 1992). The OAKHQoL self‐administered questionnaire measures the disease‐specific HRQoL of OA in five domains, namely, physical activity, mental health, pain, social support and social functioning (Rat et al. 2005). This instrument is a 43‐item scale in which each item is rated on a 1–10 Likert scale. The instrument has been validated in previous studies, including good measurement properties, valid scales and excellent mapping properties to categories of the International Classification of Functioning, Disability and Health framework specific to knee and hip OA patients (Rat et al. 2008; Goetz et al. 2011).

Secondary outcome measures included obesity measured by body mass index (BMI) and waist‐to‐height ratio (WHtR), and self‐management competency, measured by the Partners in Health (PIH) scale (Smith et al. 2017). Obesity is commonly measured using BMI, an index based on height and weight information. Being obese increases the risk of OA including the hip and knee with the knee being of greatest risk, and ‘… this occurs on a dose‐response gradient of increasing BMI’ (Reyes et al. 2016, 1870). While BMI is a parsimonious method of measuring obesity, its limitations include the inability to distinguish between fat mass and fat‐free mass (Sahafi et al. 2015). Therefore, to supplement BMI, we also measured WHtR (calculated as waist [cm]/height [cm]). Previous studies have indicated that WHtR is a more sensitive index than BMI for body fat and may correct the misclassification of BMI (Browning et al. 2010; Sahafi et al. 2015). The PIH scale is a self‐rated 12‐item questionnaire addressing chronic condition self‐management capabilities across a range of chronic conditions (Battersby et al. 2003; Smith et al. 2017). The most recent version of PIH has shown to be an internally consistent and structurally valid instrument for measuring chronic condition self‐management in a range of settings/conditions, for example, Australian general community (Smith et al. 2017), multiethnic sample of patients (Shou et al. 2024), liver cirrhosis (Ramachandran et al. 2021) and heart failure (Iyngkaran et al. 2024). The scale's validated 4‐factor structure measures self‐management concepts related to ‘Knowledge’—an individual's understanding of their health condition and treatment; ‘Partnership’—the nurse and allied health professional–patient partnership; ‘Management’—the patient's ability to manage and monitor signs and symptoms; and ‘Coping’—the patient's capacity to cope with the impact of their chronic condition on physical functioning, emotions and social relationships (Smith et al. 2019).

Outcome measures were administered at baseline, presurgery (6 months' post‐baseline) and 10 months' postbaseline visits at the RGH by the primary researcher not involved in delivering the programme. Twenty‐six patients in each group received surgery between 6 and 10 months. To maximise the return rate, questionnaires were posted or asked on the phone if participants did not attend their follow‐up appointments.

### 2.7 Ethical Considerations

Because this study was motivated by uncertainty about the clinical superiority of the intervention programme over the usual care pathway, the principle of clinical equipoise applied where participants were not being disadvantaged by assignment to either study group. The study received ethics approval from the Southern Adelaide Clinical Human Research Ethics Committee (HREC/14/SAC/414 [401.14]) committee and was registered with the Australian New Zealand Clinical Trials Registry (ACTRN12615000674538). Participants were given an information sheet regarding the study and asked to provide written informed consent at the first visit before data collection began. The research assistant (LS) was responsible for the day‐to‐day running and management of the trial and reported to the trial group members, including the chief investigator, co‐investigators and trial statistician.

### 2.8 Data Analysis

This preliminary trial was designed for 80% power to detect a medium effect size (*d* = 0.6) using a HRQoL continuous measure. For each study group, 36 participants were required. With an anticipated dropout rate of 30%, a total sample size of 94 participants was needed. This sample size met the recommended criteria that an appropriate pilot size is *N* ≥ 55 (Whitehead et al. 2016). The analyses followed an intent‐to‐treat (ITT) principle to identify any statistically significant changes in primary and secondary outcome scores across time, where all participants were compared, regardless of their adherence to or completion of the programme. We also undertook a per study‐protocol analysis to assess intervention effects when analysing data for participants with complete sets of outcome assessments. Participant baseline characteristics were reported using descriptive statistics—number, percentage, mean and standard deviation.

We used linear mixed‐effects models for the repeated continuous measures of primary and secondary outcomes. Advantages of mixed‐effects models include using all available data, not influenced by data missing at random, and permit valid statistical inferences even if some model assumptions, such as normality, are violated (Gueorguieva and Krystal 2004; Rabe‐Hesketh and Skrondal 2022). Model diagnostics included the generation of standardised residuals to investigate model assumptions such as normally distributed errors and whether the fit of each model was sensitive to unusual observations (West et al. 2014; Rabe‐Hesketh and Skrondal 2022). To visually detect any deviations from normality, we used quantile–normal plots where the tails of the distribution are emphasised (Miller Jr. 1997). Adjustment for the baseline value of each outcome variable was made by excluding the main effect of the treatment variable in the models (Twisk et al. 2018). The model was used to test for treatment group effect at the completion of the intervention and for maintenance effects; estimates were presented along with 95% confidence intervals (CIs). For any statistically significant findings in primary outcome measures, effect size statistics (Cohen's *d*) were calculated to compare within‐group 10‐month versus baseline mean observed scores (Cohen 2013). The interpretation of Cohen's d may be guided by the conventional standard of 0.2–0.49 as small, 0.5–0.79 as medium and 0.8 or above as large. Statistical analyses were conducted using Stata 16.1 (StataCorp 2019).

## 3 Results

### 3.1 Participant Flow and Recruitment

**FIGURE 1:**
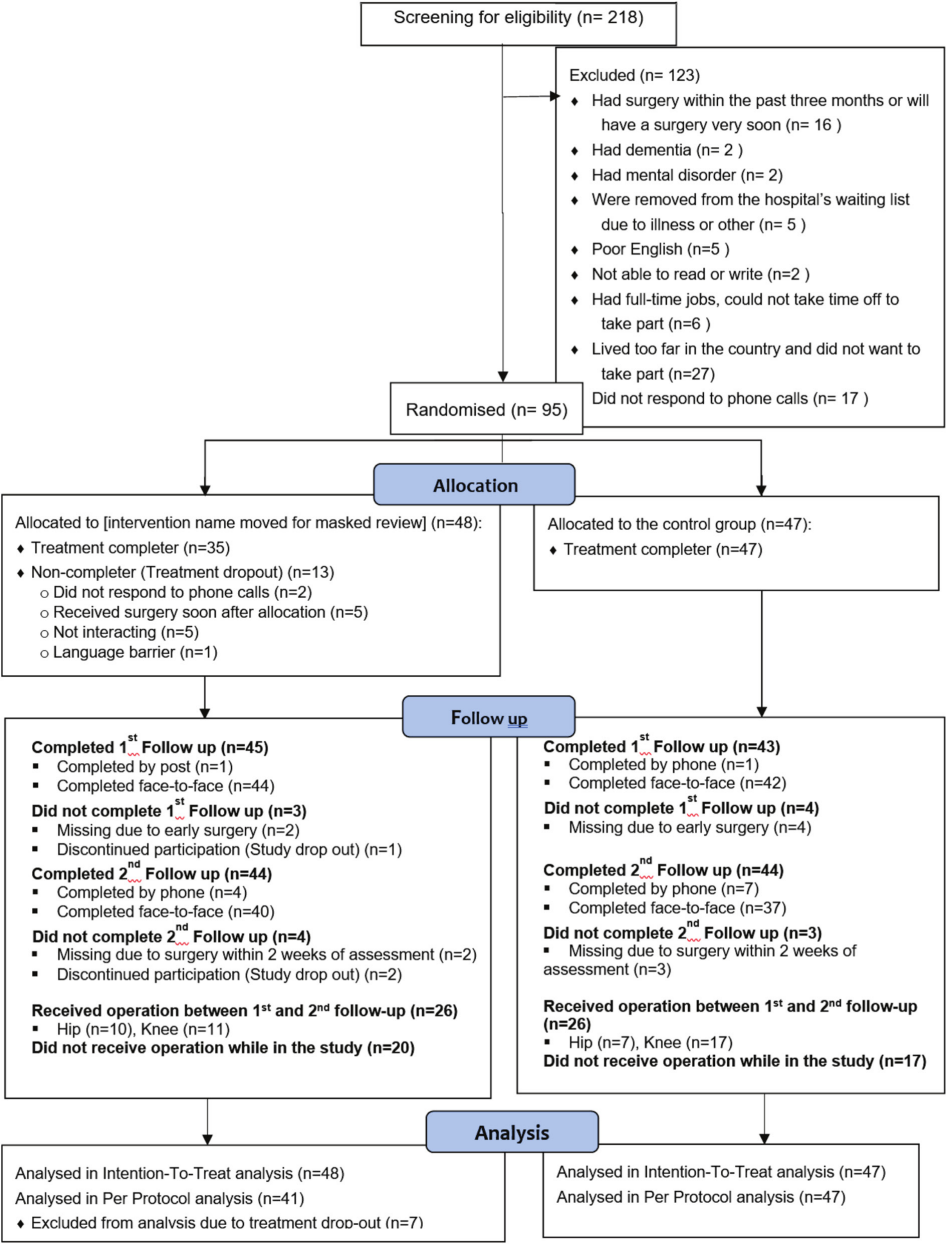
Participant flow.

Figure 1 shows the flow of participants throughout the trial. Participants were recruited from a total of 218 hip/knee OA patients on the joint replacement waiting list from July 2015 to February 2016. The main reason for patients refusing to take part in the study was living in the country and having limited access to transport to attend the study site. The most common reason for study exclusion was residential distance from the hospital and lack of easy access to transport arrangements (𝑛 = 27). A total of 95 participants (44%) consented to the study.

### 3.2 Baseline Characteristics

Baseline measurements were taken from all 95 enrolled participants before randomisation into one of two groups (48 intervention and 47 controls) using stratified block randomisation. The baseline socio‐demographic and clinical characteristics of both groups were similar (Table 1). The total SF‐36 physical summary score was similar for both groups, with 31.7 (SD = 7.2) for the control group and 31.5 (SD = 6.3) for the intervention group, both considerably lower than the South Australian population of 53.621. The mean SF‐36 mental summary score was 47.2 (SD = 12.4) for the control group and 44.2 (SD = 12.6) for the FP group (the SF‐36 mental summary score is 48.8 for the South Australian population). For OAKHQoL, there were some small to moderate differences, particularly for the subscales mental health, social activity, professional activities, spouse relations and sexual activity. We accounted for this chance discrepancy through the adjustment of baseline values for each outcome variable in the primary efficacy analysis presented in the next section.

**TABLE 1:** Baseline characteristics.

	Control group (*n* = 47)	FP group (*n* = 48)
Socio‐demographic data		
Age (years)	68.5 (8.2; range, 46–85)	67.3 (9.0; range, 48–83)
Female	29 (61.7%)	29 (60.4%)
Male	18 (38.3)	19 (39.6)
Living arrangement		
Living alone	17 (36.2%)	15 (31.2%)
Living with partner	25 (53.2%)	29 (60.4%)
Living with children	3 (6.4%)	2 (4.2%)
Other	2 (4.3%)	2 (4.2%)
Work status		
Retired/unemployed	40 (85.1%)	39 (81.2%)
Full‐time job	3 (6.4%)	4 (8.3%)
Part‐time job	4 (8.5%)	5 (10.4%)
Qualification		
Primary school	5 (10.6%)	3 (6.2%)
Secondary school	30 (63.8%)	31 (64.6%)
Undergraduate	4 (8.5%)	6 (12.5%)
Postgraduate	3 (6.4%)	4 (8.3%)
Other	5 (10.6%)	4 (8.3%)
Clinical characteristics		
BMI	37.7 (4.4)	36.8 (4.6)
WC	119.1 (14.0)	118.3 (12.1)
WHtR	0.7 (0.1)	0.7 (0.1)
%BF	40.8 (5.1)	40.7 (5.3)
SF‐36		
Physical function	29.7 (6.8)	29.5 (7.2)
Physical role	34.7 (8.5)	32.6 (7.5)
Bodily pain	32.1 (5.6)	30.8 (6.3)
General health perception	46.8 (11.2)	44.4 (9.9)
Total physical score	31.7 (7.2)	31.5 (6.3)
Vitality	40.1 (10.6)	39.7 (8.5)
Social role	36.5 (13.4)	37.6 (12.8)
Emotional role	41.1 (12.6)	36.2 (13.1)
Mental health	47.3 (11.7)	44.1 (10.4)
Total mental score	47.2 (12.4)	44.2 (12.6)
OAKHQoL		
Physical activity	35.5 (16.6)	32.1 (19.1)
Mental health	59.8 (24.0)	50.6 (26.4)
Pain	28.5 (19.5)	25.8 (22.5)
Social support	67.8 (24.6)	68.2 (20.5)
Social activity	62.6 (27.6)	55.8 (24.4)
Professional activities	61.7 (34.9)	41.7 (33.6)
Spouse relations	58.5 (33.1)	43.2 (36.9)
Sexual activity	54.8 (41.9)	38.3 (38.5)
Self‐management (PIH)	77.8 (13.9)	78.1 (10.5)
Knowledge	13.7 (2.3)	12.8 (3.5)
Partnership	29.8 (4.4)	30.2 (2.7)
Symptom recognition & management	13.9 (2.4)	13.8 (12.9)
Coping	22.3 (6.8)	21.5 (6.8)

*Note:* Data are mean (standard deviation [SD]), or *n* (%).

Abbreviations: %BF = percentage body fat; BMI = body mass index; WC = waist circumference; WHtR = waist‐to‐height ratio.

Two of the 95 participants dropped out of the study due to their unwillingness to attend the study's follow‐ups. They were both allocated to the intervention group and did not attend the first session of the intervention programme. They were both male and morbidly obese (BMIs of 52.8 and 47.7). Also, 14.6% (7/48) participants were classified as treatment noncompleters in the intervention group, having received less than one‐third of the intervention (3 or less of a possible 12 intervention phone calls). This included two females and three males who did not continue the intervention after attending the first session and two males who did not attend the first session. There were no noncompleters in the control group as the usual care was part of routine hospital care.

For FP participants, SF‐36 data were available for 89.6% (43/48) on both 6‐month and 10‐month follow‐up occasions, and 97.9% (47/48) provided at least one set of follow‐up data. For control, SF‐36 data were available for 87.2% (41/47) at both 6‐month and 10‐month follow‐up, and all participants provided at least one set of post‐baseline data. Twenty‐six participants in each of the intervention and control groups had their operation at some point after the first follow‐up and before the second follow‐up. There were no reports from either patients or staff of harms or unintended effects in each group.

### 3.3 Preliminary Efficacy of FP

#### 3.3.1 Primary Outcomes

Using all available data (*N* = 95) and adjusting for baseline values, Table 2 provides results from within group estimates (the ‘unadjusted estimate’ column) and between group comparisons (the ‘between‐group difference across time points’) using mixed‐models for SF‐36 and OAKHQoL. Quantile‐normal plots of standardised residuals for each mixed‐model indicated that predictions had distributions reasonably close to normal and therefore were good‐fitting models. For SF‐36, the FP group experienced a greater improvement in emotional role score across time (baseline to 10‐month follow‐up) as measured by SF‐36 compared with usual care (UC) at the 10% level (*p* = 0.09). Within group effect size analyses comparing observed scores at 10‐months versus baseline indicated FP participants, on average, experienced an increase (improvement) of medium effect size (Cohen's *d* = 0.61; 95% CI: 0.19–1.03) and UC small effect size (*d* = 0.17; 95% CI: −0.25 to 0.58). The width of these confidence intervals however indicated considerable variability in precision of *d* values. There were no other clinically discernible differences between groups for SF‐36 domains mental (*p* = 0.25) and physical (*p* = 0.69) and subscales.

**TABLE 2:** Changes in primary outcome between control and intervention groups across baseline, 6‐month and 10‐month time points.

	Baseline		6 months		10 months		Between‐group differences across time points	
	Unadjusted estimate (standard error [SE])		Unadjusted estimate (standard error [SE])		Unadjusted estimate (standard error [SE])			
	Control *N* = 47	Intervention *N* = 48	Control *N* = 47	Intervention *N* = 48	Control *N* = 47	Intervention *N* = 48	Adjusted estimate (95% CI)	*p* value
SF‐36 total physical	31.1 (1.1)	31.1 (0.1)	34.5 (0.9)	34.7 (0.9)	36.7 (1.1)	37.1 (1.2)	0.02 (−0.06 to 0.03)	0.686
SF‐36 total mental	47.2 (1.8)	44.1 (1.7)	44.0 (1.9)	42.2 (1.7)	45.5 (1.7)	46.0 (1.6)	0.06 (−0.04 to 0.15)	0.250
Physical function	29.6 (1.1)	28.5 (1.0)	28.8 (1.2)	31.8 (0.9)	32.9 (1.2)	34.0 (1.2)	0.04 (−0.04 to 0.12)	0.355
Physical role	34.2 (1.2)	32.1 (1.1)	36.9 (1.0)	35.9 (0.8)	38.6 (1.2)	38.4 (1.2)	0.01 (−0.07 to 0.09)	0.762
Bodily pain	31.4 (0.9)	30.1 (1.0)	34.3 (0.9)	34.0 (0.9)	36.3 (1.3)	36.9 (1.2)	0.03 (−0.06 to 0.11)	0.509
General health	46.5 (1.5)	44.5 (1.4)	47.4 (1.4)	46.0 (1.3)	48.0 (1.4)	47.1 (1.4)	0.01 (−0.06 to 0.08)	0.714
Vitality	39.5 (1.6)	39.0 (1.2)	42.2 (1.4)	41.3 (1.0)	44.0 (1.6)	42.8 (1.2)	−0.01 (−0.10 to 0.07)	0.779
Social role	36.4 (1.9)	37.4 (1.8)	33.9 (1.9)	37.2 (1.8)	36.9 (1.8)	40.4 (1.6)	0.07 (−0.03 to 0.17)	0.184
Emotional role	41.1 (1.8)	36.0 (1.7)	38.0 (1.8)	35.7 (1.7)	40.5 (1.6)	41.6 (1.6)	0.10 (−0.01 to 0.19)	0.090
Mental health	47.3 (1.7)	43.9 (1.5)	44.2 (1.8)	42.1 (1.6)	45.6 (1.8)	44.7 (1.6)	0.02 (−0.08 to 0.13)	0.647
**OAKHQoL**								
Physical activity	35.3 (2.6)	29.9 (2.8)	31.9 (2.9)	36.9 (2.7)	41.8 (3.2)	41.4 (3.4)	0.05 (−0.17 to 0.27)	0.671
Mental health	59.7 (3.5)	48.5 (3.6)	57.4 (3.6)	57.1 (3.3)	62.2 (3.5)	62.8 (3.7)	0.19 (−0.01 to 0.38)	0.066
Pain	25.0 (3.4)	23.8 (3.4)	35.3 (2.7)	35.3 (3.2)	42.2 (3.2)	42.9 (4.1)	0.06 (−0.18 to 0.30)	0.599
Social support	68.4 (3.3)	68.5 (2.7)	68.2 (2.7)	72.8 (2.2)	68.1 (3.1)	75.6 (2.6)	0.20 (0.02–0.37)	0.031
Social activity	62.6 (4.1)	53.5 (3.3)	51.4 (4.1)	59.0 (2.5)	53.6 (3.8)	63.7 (3.2)	0.31 (0.10–0.54)	0.005
Professional activity	58.9 (9.6)	36.3 (8.2)	57.6 (6.8)	48.2 (8.0)	56.8 (9.4)	56.2 (10.4)	−0.10 (−0.89 to 0.68)	0.793

For OAKHQoL Mental Health domain, FP participants experienced a greater increase (improvement) in scores at 10‐month follow‐up compared with UC; this was significant at the 10% level (*p* = 0.07). The within‐group effects were of medium size for intervention participants (*d* = 0.56; 95% CI: 0.13–0.98) and small for UC (*d* = 0.30; 95% CI: −0.12 to 0.71). There was reasonably strong statistical evidence in favour of the intervention group for improvements in sub‐scales social support (*p* = 0.03) and social activity (*p* = 0.005). For social support, the magnitude of these effects was small for FP (*d* = 0.43, 95% CI: 0.01–0.83) and UC (*d* = −0.01, 95% CI: −0.41 to 0.42). A similar magnitude of effects was found for social activity, with FP having a larger effect size (*d* = 0.47; 95% CI: 0.05–0.89) relative to UC (*d* = −0.16; 95% CI: −0.58 to 0.25).

A per protocol analysis (FP: *n* = 41; UC: *n* = 43) suggested FP participants had greater improvement in SF‐36 mental domain at the 10% level (*p* = 0.08) with small effect size (*d* = 0.23; 95% CI: −0.20—0.65) versus UC (*d* = −0.07; 95% CI: −0.50 to 0.37). Similarly, for OAKHQoL mental health, FP participants experienced a statistically significant increase (improvement) across study period compared with UC (*p* = 0.02) with medium effect size (*d* = 0.51; 95% CI: 0.07–0.95) versus UC (*d* = −0.22; 95% CI: −0.21 to 0.66). All other results were consistent with those from intention‐to‐treat analysis including statistical evidence in favour of FP for OAKHQoL social support (*p =* 0.04) and social activities (*p* = 0.004).

#### 3.3.2 Secondary Outcomes

Results from secondary outcome analyses are shown in Table 3. The WHtR outcome of obesity showed a significantly greater improvement in the intervention group compared with the control group (*p* = 0.04), but not BMI (*p* = 0.62). For self‐management capacity (PIH total score), there was no statistically significant difference between groups (*p* = 0.30); however, for subscale Coping, the FP group had a statistically significant increase (improvement) in scores (*p* = 0.01). FP participants also experienced greater improvements on the Knowledge subscale, although marginally nonsignificant (*p* = 0.07).

**TABLE 3:** Changes in secondary outcomes between control and intervention groups at baseline, 6‐month and 10‐month points.

	Baseline		6 months		10 months		Between‐group differences across time points	
	Unadjusted estimate (standard error [SE])		Unadjusted estimate (standard error [SE])		Unadjusted estimate (standard error [SE])			
	Control *N* = 47	Intervention *N* = 48	Control *N* = 47	Intervention *N* = 48	Control *N* = 47	Intervention *N* = 48	Adjusted estimate (95% CI)	*p* value
BMI	37.7 (0.7)	36.8 (0.6)	37.3 (0.7)	36.2 (0.7)	37.4 (0.8)	36.3 (0.7)	−0.01 (−0.03 to 0.01)	0.615
WHtR	0.7 (0.01)	0.7 (0.01)	0.7 (0.01)	0.7 (0.01)	0.7 (0.01)	0.7 (0.01)	−0.001 (−0.001 to −0.00003)	0.035
**PIH**								
Total PIH	78.2 (1.6)	78.3 (1.3)	80.3 (1.3)	81.6 (1.2)	81.7 (1.5)	83.7 (1.3)	0.05 (−0.04 to 0.13)	0.298
Knowledge	13.7 (0.3)	13.0 (0.4)	13.8 (0.3)	13.8 (0.4)	13.9 (0.3)	14.3 (0.4)	0.02 (−0.002 to 0.04)	0.074
Partnership in treatment	30.0 (0.5)	30.3 (0.4)	30.2 (0.3)	30.4 (0.3)	30.4 (0.4)	30.3 (0.4)	−0.004 (−0.03 to 0.02)	0.743
Recognition & management of symptoms	14.1 (0.3)	13.9 (0.3)	14.5 (0.2)	14.4 (0.2)	14.7 (0.3)	14.7 (0.3)	−0.002 (−0.02 to 0.02)	0.838
Coping	22.2 (1.0)	21.4 (0.9)	22.5 (0.9)	23.3 (0.8)	22.6 (0.9)	24.6 (0.9)	0.06 (0.01–0.11)	0.013

Abbreviations: %BF = percentage body fat; BMI = body mass index; WC = waist circumference; WHtR = waist‐to‐height ratio.

## 4 Discussion

With evidence suggesting that the prevalence of OA is rising due, in part, to the increasing prevalence of OA risk factors, including obesity and physical inactivity, and joint replacement is not a cure (Hawker 2019), there is a major need for research on self‐management for this population (Fernandes et al. 2013). The current trial provided evidence that the nurse‐led FP intervention, which is grounded in a structured collaborative process addressing behaviours of both the patient and health care provider (Lawn et al. 2009), is feasible among patients with obesity and OA awaiting arthroplasty. Furthermore, preliminary evidence suggests that the intervention may have efficacy for improving mental and social domains among patients.

Specifically, we found significant improvements on OAKHQoL mental and social domains. While a similar effect was not found for SF‐36, this may reflect that OAKHQoL is condition‐specific and had better sensitivity to change in a relatively short period of time. The study period may not have been long enough for more generic change to be detected using SF‐36 in mental and social well‐being. Also, like O'Brien et al. (2018), we found no significant differences in HRQoL pain reduction between the participants in the FP compared with usual care. However, pain coping skills can provide patients with strategies to better accept and tolerate pain. Our data showed that the intervention group significantly improved their knowledge and coping skills.

In addition to detecting preliminary efficacy, we obtained empirical evidence of key feasibility parameters that will help design a more definitive clinical trial. We screened 218 potential study participants, and 95 (44%) consented and were randomised to either FP or control group. The recruited participants were typical of the target population: the average age of Australians with OA is 65–69 years, 1.8 females for every male, excess weight being a common factor that contributes to the onset and progression of OA, and OA being the most common condition leading to hip and knee replacement surgery (Australian Institute of Health and Welfare 2024). The main reason for patients refusing to take part in the study was living in the country and having limited access to transport to attend the study site. We learnt that the initial face‐to‐face assessment could be replaced by using virtual technology or telephone, as with the follow‐ups. Apart from the initial care planning session, all intervention contacts reviewing progress and providing coaching were by phone. Given this, and the fact that most participants in our study lived far from the study location and had travel difficulties due to their physical condition, and the high attendance rate, the form of follow‐up provided a strength to the study by increasing participation.

A crucial aspect of clinical trial design is the method used to assign interventions to trial participants. We used stratified blocked randomisation with the intent to eliminate selection bias by balancing both known and unknown confounders. The baseline comparability of study groups was a good indicator that our randomisation procedure had worked, including the balance achieved on stratification variables: gender and BMI. While blocked stratification was advantageous in this current trial with a small sample size to help match baseline characteristics, a much larger trial may only require simple randomisation to achieve balance due to the law of large numbers (Broglio 2018).

For our primary analysis of outcomes, we used an ITT approach to avoid bias associated with nonrandom loss of participants (Moher et al. 2010). While over 97% of participants provided at least a 6‐month or 10‐month follow‐up assessment, up to 13% of participants did not have complete data for all assessments. To handle missing data, we used mixed models with the underlying assumption that any missing data is at least missing at random (MAR). This means the probability of missing data is dependent on observed values, but independent of unobserved values; then it is MAR (Carpenter and Kenward 2007). To understand how far the results depend on these assumptions in a large‐scale trial, the primary analysis should be accompanied by sensitivity analyses to explore how the conclusions vary over a range of different credible assumptions for the missing data (Cro et al. 2016).

## 5 Limitations

While the current trial demonstrates feasibility and acceptability and shows promise for preliminary efficacy, several limitations should be noted. The participants were recruited from a single hospital in South Australia, thus limiting generalisability of findings. A further limitation is that patients on a waiting list for joint replacement are at the most severe end of the arthritis spectrum. Their capacity to improve QoL without an operation, particularly in a 6‐ or 10‐month time frame, can be limited, especially as they have the expectation that they will receive surgery and might not be ready to make lifestyle changes. Therefore, not having long‐term follow‐ups nor having a large sample size are other limitations to this study.

The small sample size also meant our per protocol analysis was limited due to the transformation from a randomised design to observational. This could potentially result in the sample means not estimating the true average treatment effect (Hernán and Hernández‐Díaz 2012). To address this in a larger future trial with sufficiently available data, treatment effects could be considered in a counterfactual framework to mitigate selection bias by including all prognostic covariates identified at the trial design stage (Hernán and Hernández‐Díaz 2012).

## 6 Conclusion

The preliminary evidence from this study is that a self‐management support intervention programme could be a beneficial adjunct to patient care, specifically in providing improvements in mental health, coping and knowledge in obese patients with advanced hip or knee OA, and in weight loss for severely and morbidly obese patients. As widely reported (Ring 2015) mental health issues including depression are important, if not the most important, in affecting the outcome of orthopaedic surgery. Lowering BMI for severely and morbidly obese patients before surgery using the FP may improve surgical outcomes and lower the risk of complications. The current findings have the potential to inform a future, more definitive randomised controlled trial.

## Author Contributions

L.S., D.S., R.J. and M.B. contributed to the study conception and design. Material preparation and data collection were performed by L.S. Data analysis and interpretation were performed by L.S., D.S., R.J. and M.B. The first draft of the manuscript was written by L.S., and all authors commented on previous versions of the manuscript. All authors read and approved the final manuscript.

## Ethics Statement

This study was performed in line with the principles of the Declaration of Helsinki. The study received approval from the Southern Adelaide Clinical Human Research Ethics Committee (HREC/14/SAC/414 [401.14]).

## Consent

Informed consent was obtained from all individual participants included in the study.

## Conflicts of Interest

Professor Malcolm Battersby, one of the authors, has a potential conflict of interest as the lead of the team that has developed ‘The Flinders Program’, the intervention examined in this publication. We have no other conflicts of interest to disclose.

## Data Availability

The data that support the findings of this study are available on request from the corresponding author. The data are not publicly available due to privacy or ethical restrictions.
